# Caesarean section in Sudan: findings from nationwide household surveys on rates, trends, and geographic and sociodemographic variability

**DOI:** 10.1186/s12884-022-04995-3

**Published:** 2022-09-20

**Authors:** Manar E. Abdel-Rahman, Lukman Thalib, Duriya A. Rayis

**Affiliations:** 1 Department of Public Health, College of Health Science, QU Health, Qatar University, Doha, Qatar; 2 Department of Biostatistics, Faculty of Medicine, Istanbul Aydın University, Istanbul, Turkey; 3 Faculty of Medicine, University of Khartoum, Khartoum, Sudan

**Keywords:** Sudan, Caesarian section, Trend, Inequalities, Determinants, Regional variability, Area of residence, Wealth, Sociodemographic

## Abstract

**Background:**

Medically unjustifiable caesarean section (CS) deliveries have been rising rapidly in many developed countries over the last three decades. While many developing countries show rates beyond optimal levels, few poorer countries appear to have sub-optimal obstetric care in relation to essential surgeries. The objective of this study is to document the rates of CS delivery, its time trend, and geographic and sociodemographic variability in Sudan.

**Methods:**

We utilized a number of Multiple Indicator Cluster Surveys (MICS) conducted in 2014, 2010, and 2006 to quantify CS rates per 1000 live births. We also documented absolute changes in rates over three-time points and variation in CS rates across geographic regions and areas of residence.

**Results:**

Over a decade, CS rates in Sudan increased steadily from 4.3% in 2006 to 6.7% in 2010 and 9.1% in 2014. During this period, CS rates varied considerably across regions showing higher rates in the Northern region (7–25%) and lower rates in Darfur (2–3%). Urban areas experienced rapidly increasing rates (6–14%), while rural areas showed negligible changes to absolute CS rates over time (5–7%). We also found geographic regions, maternal age, maternal education, receiving antenatal care, and birth order of the child were important determinants of CS in Sudan.

**Conclusion:**

Sudan may be facing a double burden of problems associated with surgical interventions for childbirth. While the wealthier parts of Sudan are experiencing a rapid surge in CS, some poor parts of rural Sudan may not be getting the essential surgical intervention for birth when mandated. Urgent improvement to obstetric care and the development of appropriate public health interventions that focus on regional disparities are warranted.

## Background

Medically unjustifiable caesarean section (CS) deliveries have been rising rapidly in many developed countries over the last three decades [1–3]. While many developing countries show similar increasing rates of CS over the past decade, rural regions of some poorer nations still appear to have sub-optimal obstetric care in relation to essential surgeries [4]. CS is a life-saving obstetric surgery for women and newborns when certain complications occur during pregnancy or birth [5, 6]. However, when used without medical indication, the surgical procedures were shown to be associated with complications in mother and child. Some of these adverse events may even affect future pregnancies [7].

Globally, the number of babies born through CS almost doubled between 2000 and 2015—from 12 to 21% of all births, according to a series of papers published in *The Lancet* [3, 8]. About two-thirds of this increase was mainly driven by an increase in the proportion of births occurring in health institutions; the remaining one-third was attributed to an increase in CS use within health institutions [3]. In 1985, the World Health Organization (WHO) convened a group of experts to discuss the optimal level of CS and to identify ways to curb the rapidly surging rates of caesarean surgeries. Experts concluded that rates higher than 10–15% are unjustifiable.

The WHO panel met again in 2015 and concluded that CS rates are increasing steadily in many parts of the world and rates higher than 10% are not associated with reductions in maternal and newborn mortality rates [5, 9]. WHO guidance stressed on the need for a comprehensive health education, including tailored information and support about childbirth fear, pain relief, and the advantages and disadvantages of CS be provided to all women. New WHO guidance to reduce unnecessary CS made the *The Lancet* dedicate their Oct 13 issue of 2018 to discuss the issue of curbing the increasing CS rates around the world [10]. The editor and the authors discussed ways and means to reduce the CS rates, globally.

Since, the WHO alert on alarmingly increasing rates, studies from different parts of the word suggested that CS deliveries were in excess in many middle- and high-income countries, having negative implications and generating higher health care expenditure [11]. On the other hand, this life-saving surgery is still unavailable in some low-income countries [12], with rates of less than 5% potentially indicating unmet needs and potentially contributing to increased neonatal mortality [11, 13]. Despite heightened awareness created after the WHO meeting in Brazil in 1985, the re-assessment carried out in 2018 found that CS rates have increased dramatically, reaching a global rate of 18.6% of all births [1]. Some countries were experiencing CS rates far beyond the justifiable levels (e.g., 51.8% in Egypt), while rates were very low in some low-income countries (e.g., 3.0% in West Africa).

Sudan is the third-largest county in Africa, with over 43 million people. While rich in natural and human resources, poverty levels are high, particularly in rural regions [14]. This is important as the majority of the population (75%) is living in rural areas, and about two-thirds of births occur at home [15]. Although the country adopted a free-of-charge CS order in 2008, it has not been fully implemented [16, 17]. Hospital-based studies found two-fifth of deliveries in the capital city, Khartoum, and about one-fifth in the Eastern region of Sudan were CSs [18, 19]. There is however lack of nationally representative studies on this crucial subject.

A number of studies from other developing countries, showed the rural-urban divide in the CS rates as well as difference by geographical regions and wealth [20–24]. The present study aimed to document the CS rate in Sudan at the national level, and assess its changing patterns over time, across geographical regions, area of residence, and wealth. There were no previous studies that documented the nationwide rates nor could we find the assessment of changing rates of CS over time in Sudan. We envisage our findings would facilitate comparisons of CS rates in Sudan with other countries and regions and enable benchmarking in relation to the 10% increased rate referenced by the WHO. Considering the potential negative concerns of higher unjustifiable CS rates, this research also attempted to identify key determinants of CS deliveries to assist policymakers in understanding the population at high risk requiring interventions.

## Methods

### Data

We utilized secondary data, from ever-married women aged 15–49 years, extracted from three national household surveys conducted in Sudan: Multiple Indicator surveys (MICS) round 5, MICS round 4, and Sudan household survey (SHHS2) conducted in 2006, 2010, and 2014, with unweighted sample sizes of 10,776, 5646 and 5780 live births, respectively. We used the most recent MICS 2014 to assess determinants of CS, restricting the analysis to singleton and last-born births over the past 2 years preceding the survey (unweighted *n* = 5589). Multiple births (unweighted *n* = 191 births) were excluded due to their inherent higher risk of CS delivery. Anonymized data are freely available upon request and approval from the MICS program of the United Nations International Children Education Fund (UNICEF) [25], and detailed reports on survey designs, methods, and findings are reported elsewhere [25, 26].

### Variables

The main outcome variable was an event of a CS reported by the mother over the past 2 years preceding the survey. Main explanatory variables included: geographical regions (Khartoum, Northern, Eastern, Central, Kordofan, and Darfur), area of residence (urban or rural), and wealth (Richer, middle, and poorer). Wealth was measured through five quintiles of the wealth index following the MICS methodology [27, 28]. It was further categorized into three categories by combining the first and second quintile into a “poorer” category and the fourth and fifth quintile into a “richer category”.

In terms of identifying further factors associated with the CS rates, the selection of potential determinants was informed by literature [2, 29, 30]. We obtained data on birth order of the child and birth interval by merging data collected via the women questionnaire with the birth history dataset. The surveys did not collect data on ethnicity, religion or any other personal information. Although data was available on whether the CS decision was made prior to the onset of labor or not, there were no data on who made such decisions nor were there any information on the potential reasons for opting for CS.

### Statistical analysis

We computed CS rates 95% confidence intervals (95% CI) as percentages of the number of women delivering by CS divided by the number of women having live births. We computed the national CS rates for each of the three time points to compare if the rates varied over the period from 2006 to 2014. We also explored if the CS rates and the time trend is linked to the geographical regions and area of residence.

We derived the CS rates in health facilities by dividing the overall CS rate by the percentage of deliveries in health facilities extracted from the 2006 and 2014 MICS [21, 28]. Following Boerma et al. method, we computed the average annual rates of increase (AARI) in CS rates, health facility deliveries, and CS deliveries in health facilities as ln (value in 2014/value in 2006)/8 years [3]. We then computed the relative contribution of change in CS use from health facility deliveries and CS in health facilities by dividing the AARI in CS by the respective AARI in health facility deliveries and CS deliveries in health facilities [3].

Using the sample from MICS 2014, we computed CS rates among all singleton births by different subgroups. As mothers who undergo CS delivery for the first time are more likely to deliver their subsequent babies via CS, we conducted a sensitivity analysis using a sample from MICS 2014, limited only to primiparous women. We fitted logistic regression models using MICS 2014 to evaluate the CS determinants by including the main explanatory variables (area of residence, region, and wealth). We selected additional variables with *p*-values < 0.25 from the crude analyses to develop an initial multivariable model [31]. Variables were then eliminated if adjusted Wald tests were not statistically significant at *p*-values < 0.05. We assessed confounding by comparing crude and adjusted odds ratios (aOR). The variables that were found to be associated remained in the final model if the change between crude and aOR was at least 20%. Potential statistical interactions between the region and the other main explanatory variables were tested. We reported odds ratios (OR), aOR, and their 95% CI from logistic regression models.

We used Stata/MP version 16.0 in all analyses [32]. Stata *Svy* command using Taylor linearization for the variance estimation was utilized to account for the complex survey design using assigned weights, primary sampling units, and strata.

## Results

**Fig. 1:**
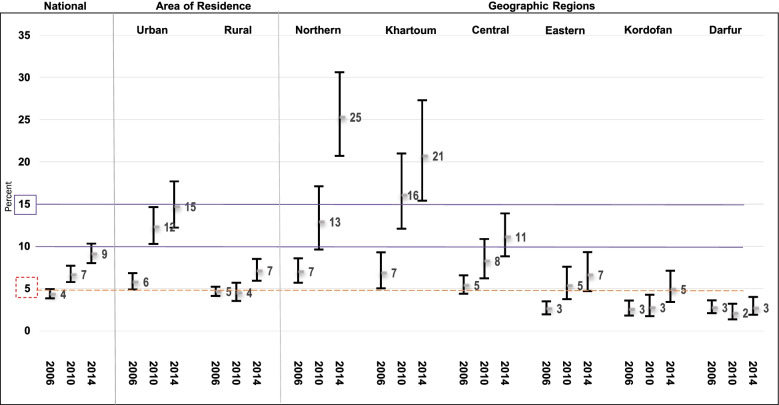
Caesarean section rate and 95% confidence intervals over time, Sudan MICS 2006, 2010 and 2014 (Unweighted *n* = 10,776, 5646 and 5780 all livebirth, respectively)

The overall CS prevalence in Sudan increased steadily from 4.3% in 2006 to 6.7% in 2010 and 9.1% in 2014 (Fig. 1) with an AARI of 9.4%. The percentages of deliveries in the health facilities were 19.9 and 28.5% in 2006 and 2014, respectively accounting for an AARI of 4.5%. Of these, CS deliveries accounted for 21.6% in 2006 and 31.9% in 2014, with an AARI of 4.9%. As such, the increase in CS (i.e., AARI = 9.4%) is approximately equally attributed to the increase in delivery occurring in health facilities (49.7%) and to the increase in CS deliveries at health facilities (52.2%).

Figure 1 illustrate that the CS rates varied considerably across regions, with a clear rural-urban divide. Over time, the highest CS rates were recorded in the Northern region (7–25%), while the lowest rates were in Darfur (2–3%) region. Over about a decade, CS rates increased rapidly in urban regions, from 6% in 2006 to 14% in 2014, while rates in the rural area remained largely unchanged at 5–7%.

In 2014, 8.9% (95% CI: 7.7–10.0) singleton births were delivered using CS in Sudan (Table 1). There was significant variability in CS rates, with urban areas experiencing a higher CS rate (14%) than rural (7%). The Northern region (25, 95% CI: 21–31%) and Khartoum (21%; 95% CI: 15–27%) had higher CS levels, while the mostly rural regions such as Kordofan (4.9%; 95% CI; 3.4–7.1%) and Darfur (2.5%; 95% CI: 1.9–4.0%) showed lower CS level. As expected CS rates decreased considerably by wealth from 24.6%; 95% CI: 20.9–28.8 among women from the richest households to 2.6%; 95% CI: 1.6–4.0 among those from the poorest (Table 1).

**Table 1:** Caesarean section (CS) rates and their 95% confidence intervals, Sudan MICS 2014

	**All singleton births**		**Primiparous**	
	**n (%)**	**CS % (95% CI)**	**n (%)**	**CS % (95% CI)**
**Total**	5509^*^ (100.0)	8.9 (7.7, 10.0)	910^**^ (100.0)	12.9 (9.4, 15.2)
**Area of residence**				
Rural	4049 (73.5)	6.7 [5.6,8.2]	691 (75.9)	10.6 [7.7,14.4]
Urban	1460 (26.5)	14.4 [11.9,17.3]	219 (24.1)	16.4 [11.2,23.4]
**Region**				
Khartoum	667 (12.1)	20.4 [15.2,26.9]	101 (11.1)	25.3 [12.6,44.3]
Northern	242 (4.4)	25.1 [20.7,30.2]	59 (6.5)	37.8 [30.4,45.8]
Eastern	585 (10.6)	6.0 [4.3,8.4]	98 (10.7)	5.4 [2.5,11.2]
Central	1597 (29)	10.7 [8.4,13.4]	321 (35.3)	12.7 [8.4,18.7]
Kordofan	865 (15.7)	4.7 [3.2,6.9]	118 (13)	7.1 [2.4,19.1]
Darfur	1553 (28.2)	2.6 [1.7,3.9]	213 (23.4)	3.2 [1.5,6.8]
**Wealth index categories**				
Richer	1899 (34.5)	17.7 [15.3,20.4]	343 (42.1)	21.2 [16.0,27.7]
Middle	1166 (21.2)	6.6 [4.7,9.2]	216 (23.8)	10.2 [6.3,16.3]
Poorer	2443 (44.4)	2.9 [2.2,3.9]	350 (38.5)	4.0 [1.9,8.2]
**Maternal age in years**				
15–24	1502 (27.3)	7.3 [5.6,9.5]	643 (70.6)	8.7 [6.0,12.5]
25–34	2730 (49.5)	8.4 [7.1,10.0]	241 (26.4)	19 [14.0,25.2]
35+	1277 (23.2)	11.2 [8.9,14.0]	27 (2.9)	28 [13.0,50.1]
**Mother’s education**				
None	2207 (40.1)	2.7 [2.0,3.7]	227 (25)	4 [2.1,7.6]
Primary	1980 (35.9)	7.0 [5.9,8.4]	318 (35)	9.4 [6.2,14.0]
Secondary+	1322 (24)	21.5 [18.5,24.8]	364 (40)	19.3 [14.7,24.8]
**Number of times received antenatal care**				
0	1068 (19.7)	1.6 [0.8,3.0]	92 (10.3)	1.2 [0.2,7.9]
1–3	1566 (28.9)	4.5 [3.5,5.8]	256 (28.5)	5.6 [3.1,9.9]
4+	2782 (51.4)	14.1 [12.3,16.2]	550 (61.2)	16.9 [13.0,21.7]
**Child’s sex**				
Male	2822 (51.2)	9.2 [7.8,10.8]	471 (51.8)	12.2 [8.9,16.6]
Female	2686 (48.8)	8.3 [6.9,10.0]	438 (48.2)	11.8 [8.6,15.9]
**Child birth-order**				
1	910 (16.5)	12.0 [9.4,15.2]		
2–3	1642 (29.8)	11.6 [9.6,14.1]		
4–6	1951 (35.4)	6.4 [5.2,8.0]		
7+	1006 (18.3)	5.8 [3.9,8.3]		
**Size of child at birth**				
Larger than average	722 (13.3)	9.3 [6.8,12.5]	107 (11.7)	18.4 [10.6,30.0]
Average	2835 (52.4)	9.8 [8.3,11.7]	489 (53.7)	10.5 [7.6,14.2]
Smaller than average	1856 (34.3)	7.1 [5.7,8.7]	304 (33.4)	12.6 [8.2,18.9]

Unweighted sample size: *n* = 5589^*^, *n* = 919^**^

**Fig. 2:**
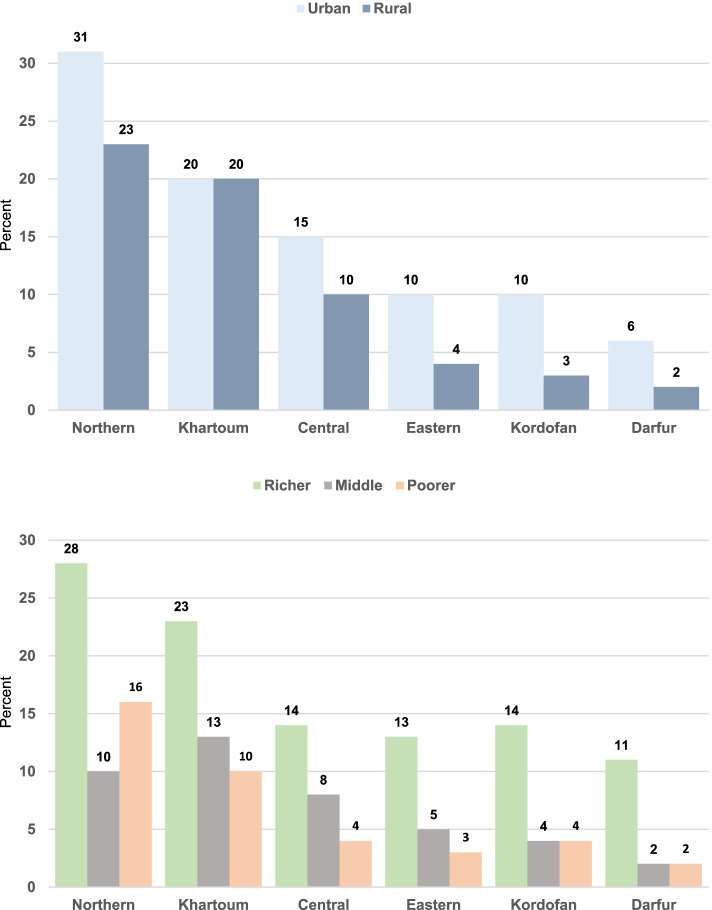
Caesarean section rates by region and area of residence and by region and wealth, Sudan MICS 2014 (unweighted *n* = 5589 singleton births)

In terms variations within each region, the CS rates in 2014, were higher among mothers living in urban areas and in richer household compared to rural and poorer ones, respectively (Fig. 2). In 3–4 out of the six regions, CS rates were considerably low (below 5% level) among mothers in rural areas and those in poorer households—for example, in Darfur, Kordofan and the Eastern regions.

Based on the 2014 survey, most births occurred at home (72%); the remaining were mostly at public facilities (26%) and only 1.7% of births occurred in private hospitals (data not shown). Among all CS deliveries, only 8% were delivered in private facilities. Significant variability was observed when assessing the association between place of delivery (private or public) and area of residence. As expected, a higher percentage of CSs were delivered in private sector in urban areas (15%) compared to rural areas (4%) were delivered in private sector (*p*-value = 0.0028) (data not shown).

In terms of who and when the CS decision were made, data were only available to indicate if the CS decisions were made before or after the onset of labor. A majority (68%) of CS decisions were made before the onset of labor. This however did not vary significantly across rural (64%) and urban (72%) settings (*p*-value = 0.097) nor were there any regional variability that is considerable (*p*-value = 0.690) (data not shown).

Further, sensitivity analyses restricted to primiparous women showed a slightly higher overall CS rate (12.9%) compared to the full sample (8.9%) (Table 1). Determinants that were associated with higher rates of CS were similar among all mothers and those delivering for the first time. As expected, maternal age and the size of child at birth were associated with higher CS rates in primiparous women. Thus, overall the results from the full sample do not seem to be biased due to repeated caesarean deliveries.

Crude ORs and and aORs and 95% CI from logistic regression models using MICS 2014 estimating the likelihood of a woman having CS by potential determinants are displayed in Table 2. The main explanatory variables (area of residence, region, and wealth) were all significant in the crude analysis. As interactions between region and area of residence and region and wealth were either non-significant (*p*-value = 0.394) or borderline significant (*p*-value = 0.053), respectively, none were included in the final multivariable model. This adjusted model showed that higher maternal age, higher education, and receiving ANC, and lower child birth order were important determinants of CS in Sudan. Additionally, higher CS rates were seen in urban areas compared to rural (aOR = 1.18; 95% CI: 0.82, 1.69) and among women living in richer households (for example (aOR richer vs poorer = 0.87; 95% CI: 0.53, 1.43). However, adjusting for other variables, regional variability remained the most prominent determinant that may explain the variability in CS rates with area of residence and wealth both being insignificant. Compared to the capital Khartoum, aORs of CS were significantly different in all regions except the central region. The aOR of CS was higher in the Northern region compared to the Khartoum (aOR = 1.71; 95% CI: 1.05, 2.79), while Kordofan and Darfur had significantly lower aORs than Khartoum with mothers in Darfur considerably least likely to deliver via CS (aOR = 0.31; 95% CI: 0.17, 0.57) compared to those living in Khartoum.

**Table 2:** Crude and adjusted odds ratios and 95% confidence intervals from logistic regression modeling the odds of caesarean section, Sudan MICS 2014 (unweighted *n* = 5589)

	**Crude analysis**		**Adjusted analysis**	
	**OR % (95% CI)**	*p*-value^*^	**aOR % (95% CI)**	*p*-value^*^
**Area of residence**		**< 0.001**		**0.368**
Rural (Reference)	1.00		1.00	
Urban	2.3 (1.71,3.21)	< 0.001	1.18 (0.82,1.69)	0.368
**Region**		**< 0.001**		**< 0.001**
Khartoum (Reference)	1.00		1.00	
Northern	1.30 (0.81,2.00)	0.230	1.71 (1.05,2.79)	0.032
Eastern	0.21 (0.11,0.41)	0.000	0.58 (0.34,1.01)	0.055
Central	0.50 (0.31,0.70)	0.001	0.96 (0.58,1.59)	0.878
Kordofan	0.21 (0.11,0.31)	0.000	0.53 (0.30,0.96)	0.035
Darfur	0.11 (0.11,0.21)	0.000	0.31 (0.17,0.57)	< 0.001
**Wealth index categories**		**< 0.001**		**0.650**
Richer (Reference)	1.00		1.00	
Middle	0.33 (0.22,0.48)	< 0.001	0.82 (0.53,1.26)	0.360
Poorer	0.14 (0.10,0.20)	< 0.001	0.87 (0.53,1.43)	0.569
**Maternal age in years**		**0.041**		**< 0.001**
15–24 (Reference)	1.00		1.00	
25–34	1.21 (0.90,1.61)	0.311	1.42 (1.01,1.99)	0.042
35+	1.61 (1.11,2.30)	0.013	2.75 (1.71,4.43)	< 0.001
**Mother’s education**		**< 0.001**		**< 0.001**
None (Reference)	1.00		1.00	
Primary	2.71 (1.90,3.80)	< 0.001	1.67 (1.17,2.37)	0.004
Secondary+	9.81 (6.91,14.00)	< 0.001	3.3 (2.21,4.93)	< 0.001
**Number of times received antenatal care**		**< 0.001**		**< 0.001**
0 (Reference)	1.00		1.00	
1–3	2.91 (1.40,6.00)		1.81 (0.86,3.81)	0.119
4+	10.2 (5.20,19.9)		4.42 (2.14,9.14)	< 0.001
**Child birth-order**		**< 0.001**		**0.002**
1 (Reference)	1.00		1.00	
2–3	1.00 (0.7,1.3)	0.834	1.01 (0.69,1.48)	0.966
4–6	0.50 (0.41,0.70)	0.000	0.52 (0.33,0.83)	0.006
7+	0.41 (0.31,0.70)	0.002	0.49 (0.26,0.94)	0.032
**Previous birth interval**		**0.009**		
First birth (Reference)	1.00			
< 2 years	0.61 (0.41,0.81)	0.001		
2 years	0.61 (0.41,0.9)	0.004		
3 years	0.81 (0.5,1.21)	0.204		
4+ years	0.81 (0.5,1.11)	0.163		
**Child’s sex**		**0.372**		
Male (Reference)	1.00			
Female	0.9 (0.7,1.11)	0.372		
**Size of child at birth**		**0.049**		
Larger than average (Reference)	1.00			
Average	1.11 (0.70,1.50)	0.729		
Smaller than average	0.70 (0.50,1.11)	0.125		

^*^p-values in bold font are global Wald test *p*-values

## Discussion

To the best of our knowledge, this is the first study to estimate the level of CS delivery in Sudan based on population-based data. Although the national averages of CS rates in Sudan were lower than the global average, the CS rates increased steadily from 4.3% in 2006 to 9.1% in 2014, with substantial regional variations. Urban areas recorded a rapid increase in CS rates, while rates remained unchanged in the rural and relatively poor regions.

Sudan can be said to be experiencing a double burden of problems associated with CS interventions. While remarkable increases were noted in urban Sudan in particular among the richer and educated households, rural Sudan and poorer households experienced lower rates of CS. Some may argue that in the midst of the global surge of CS, lower surgical interventions for deliveries should not be of major concern. However, as concluded by Betran et al. in their systematic review, lower CS rates, below 9%, could be an indication of sub-optimal maternal healthcare and other socioeconomic levels [12].

More critically, the rapid increase in CS in urban Sudan could present significant public health implications. Despite heightened awareness created by WHO in 1985, the subsequent re-assessment carried out in 2018 found a dramatic increase in CS rates in many parts of the world, with a global average CS rate reaching 18.6% of all births [1]. High CS rates highlight the extent of the problem that shocked the international public health community [33]. There is mounting evidence that documents the negative implications of excess CS deliveries. Economic analyses pointed towards higher expenditure at individual and national levels due to unnecessary CS interventions [11].

CS rates in Sudan (9%), as documented in this study, appear to be similar to those reported in some other countries like Dijbouti (11%), lower than the Middle East and North Africa (MENA) rate (29.2%) but higher than Eastern and Southern Africa rate (6%) [3, 12, 34]. Such comparisons should be understood in the light that CS rates in Sudan maybe be under-reported due to the collapsing health care system in the country [17].

The increasing rates of CS over time documented in this study are somewhat concordant with the global trend and patterns observed in many other countries [1–3]. However, the AARI in Sudan (9.3%) during 2006–2014 is higher than the global AARI (3.7%) [3]. Although there is no published national data from Sudan beyond 2014, it would not be surprising to find that the CS rates have escalated further. The Federal Ministry of Health in Sudan is responsible for guidelines and protocols aiming to reduce CS. To the best of our knowledge, there is no documented intervention in place to reduce CS rates in Sudan, particularly with the increasing health challenges after the recent political changes in the country [35].

The study showed that the increase in CS rate is equally attributed to both the increase in deliveries in health facilities and to the increase of CS in health facilities. The increase of CS in health facilities varies largely between countries. Contrary to our results, the increase in deliveries in health facilities in the MENA region contributed more to the increase in CS rate than the increase in CS in health facilities [3]. The reason might be attributed to the high cost of surgeries and mothers’ reliance on out-of-pocket expenses in Sudan [17, 36].

In addition to regional variability, maternal age, maternal education, ANC visits, and child birth order were significant determinants of higher rates of CS in Sudan. Those determinants that led to the global surge in CS may also be driving the increase in CS among mothers from higher socioeconomic classes. Similar results are observed in several low-income countries, where CS rates were low—for example, in some countries in sub-Saharan Africa among rural and poorer women [4]. In other studies, poverty was similarly associated with lower CS rates, as in our study [37, 38]. Although in our final adjusted model, area of residence and the wealth index were not significant, the six geographical regions were strongly significantly related to CS. Others studies found similar variations in CS rates by geographic regions [20, 22, 23]. Our study sample showed that about three-quarters (74%) of the population lives in rural areas. About three-quarter (76%) of the population in Khartoum is urban while regions like Kordofan and Darfur are predominantly rural (80% each of their population). Additionally, wealth varied considerably by region —for example, 74 and 81% of the study population in Kordofan and Darfur, respectively are in the poorest wealth category while 81 and 83% of those living in Khartoum and the Northern region are in the richest. As in other studies, it is expected that urban and richer women have better access the healthcare system compared rural and poorer women [39]. It is thus clear that variation in wealth and area of residence among regions may be a possible explanation to higher CS rates in some regions like Khartoum and the Northern region and lower rates in other regions like Kordofan and Darfur. As such the regional variability in CS rates across these six regions played a role in reflecting the wealth and rural-urban divide in CS experience.

Lower CS rates were also attributed to weak health care infrastructure, availability of and poor access to surgical care and emergency obstetric service [17, 40, 41]. This is compatible with our findings showing higher educated mothers experiencing higher CS rates. Better education is associated with more exposure to health information, access to health facilities, better health-seeking behavior. Mothers’ education is also associated with a higher socioeconomic status that enables them to pay for their surgeries. Such an explanation is likely the case in Sudan, given its minimal national health insurance coverage [17]. Such findings are in concordance with other studies [29, 42, 43].

Consistent with other studies, our results found lower CS rates with suboptimal ANC utilization [2, 44]. It would be alarming if the observed association is more driven by the lack of identification of clinical indications for mothers who do not attend ANC. Such situations would potentially lead to poor outcomes, including maternal and infant morbidity and mortality. Woman’s attendance to ANC in private health facilities was linked to increased probability for her to deliver by CS [3, 29]. In this study, as most of the CS deliveries were in public health facilities, attendance of ANC in private health may not be ruled as an explanation.

Our findings are significant, given the paucity of data on the CS utilization patterns in Sudan. Our reports should provide some important baseline data for the public health community to address the double burden problem of surgical interventions for childbirth in Sudan. Although the country has a full package of strategic plans, policies, and new initiatives to promote maternal health, this study highlights potential drawbacks in implementation [17, 45]. attention to this important issue and prioritize their efforts in directing effective utilization of resources. Findings from this study may motivate the initiation of more studies to assist in directing action plans of the Sudan National Health Policy 2017–2030 in the prioritization of maternal care services, particularly in specific regions in the country.

The main strength of this study is the national representation of its data over time, allowing for observing reliable regional estimates. However, our findings should be interpreted in light of some limitations. Some variables rely on the recall of past events. Although it is less likely that mothers forget their major experience of such surgery, they would sometimes misreport other information like ANC. Although in more than two-thirds of the study sample, a decision was made to perform CS before the onset of labor, no information was collected on the reason for this decision. Such information might have shed more light on the contribution of women’s preferences to the increase in CS rates [46].

## Conclusion

Findings from this study indicate that Sudan is faced with a double burden of problems associated with surgical interventions for childbirth. While the wealthier parts of Sudan are experiencing a rapid surge in CS, some poor parts of rural Sudan may not be getting the essential surgical intervention for birth when mandated. This point is critical if future studies confirm that higher neonatal mortality may be partly attributable to the lack of surgical interventions. Focusing on the availability and provision of emergency obstetric care in health facilities and expanding midwifery training should improve maternal health outcomes in Sudan. The appropriate provision of these services is dependent on the whole health care system in Sudan, which requires significant improvements. There is a need for further in-depth studies to better monitor and assess inequalities in CS rates in Sudan and develop appropriate public health interventions that include curbing the unnecessary CS interventions while providing it to the poor when it is life-saving.

## Data Availability

The datasets used and analyzed during the study may be obtained with permission from the Multiple Indicator Cluster Survey program website (http://mics.unicef.org/) upon registration to request the data and approval from UNICEF.

## Abbreviations

CS: Caesarean Section
ANC: Antenatal Care
NNRI: Average Annual Rate of Increase
MICS: Multiple Indicator Cluster Survey
SHHS: from Sudan Household Survey
